# Teachers’ demographic and occupational attributes predict feelings of hopelessness during the COVID-19 pandemic

**DOI:** 10.3389/fpsyg.2022.913894

**Published:** 2022-11-04

**Authors:** Farshad Ghasemi

**Affiliations:** Faculty of Humanities and Social Sciences, Urmia University, Urmia, Iran

**Keywords:** hopelessness, perceived social support, occupational attributes, public school teachers, COVID-19 pandemic

## Abstract

The outbreak of the COVID-19 pandemic has resulted in many emotional consequences for teachers, including feelings of isolation, loneliness, and hopelessness. However, evidence on the prevalence of hopelessness and the associated factors in teachers during the pandemic is limited. The purpose of this research was to examine the prevalence of hopelessness in public school teachers and identify risk factors associated with it. A sample of 168 teachers aged 25–49 years participated in the study by completing the Socio-Demographic Questionnaire, the Beck Hopelessness Scale (BHS), and the Multi-Dimensional Scale of Perceived Social Support (MSPSS). The results revealed a moderate level of experienced hopelessness in teachers. Hopelessness prevalence was also significantly different across teacher gender (males = 79%), age groups (>40 = 77%), socioeconomic status (poor socioeconomic status = 70%), educational level (high school teachers = 79%), professional experience (experienced teachers = 82%), and perceived social support (low perceived social support = 79%). The results of a logistic regression analysis confirmed the effects of these demographic and occupational attributes on hopelessness by explaining ~71% of the variance in hopelessness feelings. Higher odds ratios were associated with age, socioeconomic status, and perceived social support, signifying the prominence of these factors in predicting hopelessness. The study contributes to identifying and screening teachers at risk of hopelessness in public schools and recommends promoting collegial/superior support as well as a positive school climate as the protective factors against hopelessness.

## Introduction

The teaching profession has been regarded to be a highly stressful profession with significant attrition rates due to high emotional involvement and heavy workloads (Skaalvik and Skaalvik, 2011; Harmsen et al., 2018; Ghasemi et al., 2022). Evidence suggests that there has been a significant shift in teachers’ internalizing symptoms and coping mechanisms during the COVID-19 pandemic compared to the pre-pandemic era upon experiencing new demands (Ghasemi et al., 2022). With the outbreak of the COVID-19 pandemic, new risk factors (e.g., isolation and lockdown, social and physical distancing, loss of employment, and income reduction) may threaten teachers’ emotional and mental health (Baker et al., 2021). Common consequences of such threats for teachers were heightened stress, anxiety, and depression (Santamaría et al., 2021), which may have been caused by restricted social interactions and support (Tull et al., 2020). In other words, loneliness, isolation, and lack of social support have been found to be positively associated with mental disorders (e.g., depression and hopelessness), undermining individuals’ performance (Tull et al., 2020; Wootton et al., 2022).

In addition to marked increases in stress and depression, there were also reports of high levels of hopelessness during the pandemic (Holman et al., 2020; Wootton et al., 2022). Evidence suggests that hope has significant direct effects on one’s psychological health and subjective well-being (Yıldırım and Arslan, 2022). Therefore, hopelessness could be a critical risk factor for individuals’ mental health, initiated by the negative beliefs resulting from decreasing positive expectations about the future. According to Beck’s cognitive model of hopelessness, hopelessness, revealing a psychological situation, is the pessimism in the individual’s perspective toward life and the future (Beck et al., 1976). Such negative beliefs might urge an individual to develop suicidal ideation and behaviors.

There appear to be several contributing risk factors to hopelessness. For instance, individuals with affective temperaments (e.g., anxious, depressive, and irritable temperaments) may be particularly at risk for hopelessness and suicidal behavior (Baldessarini et al., 2017). Based on the learned hopelessness theory of depression (Abramson et al., 1989), there is a causal chain beginning with the negative life events (e.g., COVID-19) influenced by situational cues (e.g., negative feedback) and inferential styles (i.e., internal vs. external) and ending with hopelessness deficits (e.g., passivity, motivational and emotional deficits, negative cognitions). In particular, hopelessness deficits may also include lowered self-esteem, which is associated with low perceived social support and interpersonal relationships (see Kleiman and Riskind, 2013). Therefore, individuals with low self-esteem have low perceived social support, which may increase the levels of hopelessness feelings (Cakar and Karatas, 2012). This model of hopelessness offers a comprehensive causal pathway that ends in the development of hopelessness. This model has also been adapted to academic settings (Au et al., 2009) by discussing the causal relations between hopelessness and academic risk factors (e.g., academic failures, academic attributional style, and contextual factors). The adapted model could also be applied to teachers. For instance, a teacher exposed to prolonged negative feedback on the part of his/her colleagues, administrators, parents, or students may develop uncontrollability cognitions, implying that negative outcomes are uncontrollable. These cognitions refer to the attributional style of the teachers in comprehending their failures, which may lead to hopeless behaviors if failures are attributed to stable and global causes (e.g., lack of teaching competence). Such attributions, accompanied by lowered self-esteem and contextual factors (e.g., lack of appreciation, lack of collegial support, and unrealistic expectations), may diminish teachers’ perceived social support and impair their ability to demonstrate help-seeking behaviors, resulting in teacher hopelessness (see Au et al., 2009; Cakar and Karatas, 2012).

**Figure 1:**
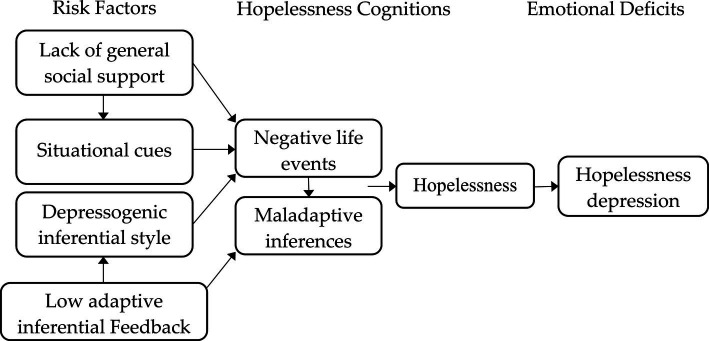
The expanded hopelessness theory of depression (based on Panzarella et al., 2006).

Figure 1 depicts an expanded hopelessness theory of depression (Panzarella et al., 2006), which examines the role of social support in the etiological chain leading to hopelessness and depression. This theory demonstrates the mechanisms by which lack of social support and low adaptive inferential feedback may influence the onset, maintenance, or prevention of hopelessness and depression. First of all, social support could reduce (a) the severity of stressful events that one may experience, (b) the cognitive vulnerability to depression using positive inferential styles, and (c) the probability of making maladaptive inferences regarding negative life events. Additionally, social support could affect the development and maintenance of a depressogenic inferential style, which refers to consistent attribution of negative life events causes to stable/global factors (Abramson et al., 1989). This cognitive style, as a risk factor for hopelessness, may increase the probability that one would make negative inferences in the face of a stressful situation. However, by providing feedback inconsistent with depressogenic inferences, adaptive inferential feedback may buffer against a negative inferential style.

The buffering effects of social support may occur either after a potentially stress-inducing event and prior to experiencing stress by affecting event appraisal, or after experiencing stress and before developing hopelessness by influencing appraisal of coping abilities or resources. Individuals in the support network may offer adaptive inferences in these situations to attribute the cause of the negative event to unstable, specific factors rather than to stable, global factors. As the opposite of depressogenic inferences, adaptive inferences by a colleague could help a teacher to ascribe a failure in the classroom to the lack of prior preparation rather than to the lack of competence in teaching (global/stable factor). In other words, the offered adaptive inferences may prompt the individual to reappraise his/her cognitions regarding the situation, resulting in the modification of the original maladaptive inference or reducing its severity and thereby, the probability of experiencing feelings of hopelessness. The most common types of social support associated with adaptive inferential feedback are informational and emotional support, which reduce one’s vulnerabilities to depressogenic inferences by providing feedback about stressors and individuals’ feelings and appraisals (Panzarella et al., 2006). Therefore, social support could play a significant role in buffering against hopelessness and depression and should be further studied during the COVID-19 pandemic when there were rigid social distancing rules, limiting perceived social support during a crisis (Tull et al., 2020; Ferber et al., 2022; Ghasemi et al., 2022).

Based on a recent study (Wootton et al., 2022) examining the level of hopelessness and its association with coping mechanisms (i.e., (dys)functional coping), there was an increase in the prevalence of hopelessness during the pandemic. Additionally, they found that dysfunctional coping was associated with heightened hopelessness, indicating the significance of coping mechanisms in controlling hopelessness feelings. Participants of the study also reported seeking support from family, friends, and mental health practitioners to manage their emotions and pandemic-related stressors. Therefore, seeking social support as a functional coping strategy has the potential to help teachers with emotional and mental problems and foster hope and optimism about the future (Ghasemi, 2022b). In other words, to deal effectively with hopelessness, teachers usually seek social support from family, friends, and/or therapists, which is considered an approach or functional coping. However, they may also utilize dysfunctional coping mechanisms (e.g., negative self-talk and self-blame), resulting in the sustenance of the disorder and/or deteriorating their mental health (Ghasemi et al., 2022; Ghasemi, 2022b). Social support also mediates the effects of life satisfaction and hopelessness on health-risk behaviors in academic settings, suggesting the importance of perceived social support in maintaining one’s health (Lai and Ma, 2016). According to a recent research study (Zuo et al., 2021) with healthcare workers during the pandemic, perceived social support negatively predicted hopelessness and can work as a psychological protective factor for alleviating it. Therefore, increasing one’s social support could be beneficial to his/her mental health and hopelessness feelings during a crisis.

Hopelessness has also been investigated in terms of demographic attributes. Hamzaoglu et al. (2010), examining the prevalence of adult hopelessness, found higher rates of hopelessness in males (35%), literates (60%), rural workforce (50%), and those with perceived bad health (59%), demonstrating the significance of demographic characteristics, social class, and perceived health in the rates of hopelessness feelings. However, the prevalence of hopelessness was higher for females and those with increased income among healthcare workers during the pandemic (Akova et al., 2022). In a general population sample of adults, the relative risk for stable hopelessness in unemployed men was found to be 7.2, and the risk was higher for women with a poor financial situation (3.8) than men (3.5; Haatainen et al., 2003). Therefore, it appears that hopelessness risk factors across demographic attributes may vary based on investigated population, indicating the need to conduct studies with different populations to examine the effects of various factors. The evidence regarding the prevalence of hopelessness in teachers is also limited, and further investigations regarding how hopelessness severity may vary for this population across gender, socioeconomic status, and education are required. As the literature on hopelessness and depression (e.g., Beekman et al., 1999; Haatainen et al., 2004; Hamzaoglu et al., 2010) indicates higher prevalence rates of these mental health issues for females, older people, and those living under adverse socioeconomic circumstances, we expect to find similar results for teachers due to the high association between hopelessness and depression (Au et al., 2009).

### Current study

Despite studies investigating feelings of hopelessness in teachers and the association of hopelessness with loneliness, self-esteem, job satisfaction, depression, and mental health (e.g., Haatainen et al., 2004; Chang et al., 2010; Cakar and Karatas, 2012; Balat et al., 2019; Huang, 2022), few studies attempted to determine the prevalence of hopelessness in school teachers to understand how it differs in terms of demographic and occupational attributes. In particular, there is limited evidence available on how it is associated with social support and the extent to which social support, as well as demographic attributes, could account for teacher hopelessness. Accordingly, this study aimed to (a) investigate the rates of hopelessness feelings in teachers during the pandemic, (b) understand how it is associated with teachers’ diverse demographic and occupational attributes, and (c) explore the extent to which these attributes account for the variations in hopelessness. We expected to find a high prevalence of hopelessness feelings in teachers due to the severe emotional and professional consequences of the pandemic for teachers in Iran (Ghasemi et al., 2022). Given the significance of the associations between hopelessness and demographic characteristics of individuals in the literature, we hypothesized to find similar results regarding such associations. Regarding the last aim, we hypothesized that teachers’ demographic and occupational attributes predict their feelings of hopelessness due to the significance of such attributes in accounting for well-being, perceived social support, emotional experiences, and coping mechanism.

Findings from the current study can help our understanding of the prevalence of hopelessness in schools and contribute to screening and identifying at-risk teachers. Additionally, studying the significance of social support in alleviating hopelessness symptoms could inform public health efforts to provide collegial/superior support for at-risk teachers by engaging them in functional coping behaviors (e.g., effective help-seeking behaviors). In other words, this inquiry could help the policymakers, administrators, school psychologists, and teachers understand possible hopelessness risk factors to promptly diagnose and intervene to effectively treat it.

## Materials and methods

### Research design

Since this study examines the prevalence of experienced hopelessness in teachers and is an attempt to understand individual differences based on demographic and occupational attributes, a cross-sectional design was used.

### Participants and research context

The working context of the research consists of secondary schools in Tehran, Iran. The education system in Iran is centralized and divided into K-12 settings with primary and secondary schools. Besides public schools, parallel private schools with a similar educational system and higher academic qualities are also available. Unlike private schools, where teachers usually deal with students with high socioeconomic status and receive high salaries, teachers in public schools receive low salaries and face students with disruptive behaviors and low socioeconomic status, which may give rise to extra pressure, stress, and/or anxiety (Ghasemi, 2022b).

A total of 203 teachers working in secondary public schools in Tehran were invited to participate in this research based on the results of a power analysis using G*Power 3.1.9.7 (Erdfelder et al., 2009). The projected sample size needed was *n* = 153 based on a small effect size of *f* = 0.15, *α* = 0.05 and power (1 − *β*) = 0.95. We had to use convenient sampling to invite the interested teachers to participate in the current study due to the consequences of the pandemic (e.g., social distancing, school closure, and remote working of teachers). All the invited teachers were briefed about the voluntary participation and study procedure by providing instructions and information regarding the study in the group channel. To be eligible to participate in the study, teachers were required to (a) have more than a year of teaching experience and (b) teach at public schools. The exclusion criteria were: (a) having less than a year of teaching experience, (b) working at private schools, and (c) attending any concurrent psychological intervention. Of the 203 teachers, 35 (17%) declined to participate in the current study, reducing the sample size to 168 (83%) teachers. Therefore, the final study sample was 168 teachers working in secondary public schools (i.e., middle and high schools) in Tehran, who were recruited by a non-probability convenient sampling method. The relevant frequencies and distributions of the demographic and occupational attributes of the participants are demonstrated in Table 1. Participating teachers were categorized based on their expertise (novice: ≤3 years; experienced: >3 years), guided by the research literature on teaching expertise. Socioeconomic status data were calculated based on the participants’ monthly income and living costs by considering their occupation and education. Based on these evaluations, most of the participants were rated to have low socioeconomic status.

**Table 1:** Teachers’ demographic and occupational attributes (*N* = 168).

Attributes	Group	*n*	%
Age	25–30	70	41.7
	30–35	44	26.2
	35–40	32	19.0
	> 40	22	13.1
Gender	Male	92	54.8
	Female	76	45.2
Education	B.A.	103	61.3
	M.A.	44	26.2
	Ph.D.	21	12.5
Education Level	Middle school	97	57.7
	High school	71	42.3
Socioeconomic status	LowMiddle	8958	53.034.5
	High	21	12.5
Professional experience	Novice	101	60.1
	Experienced	67	39.9
Total Participants		168	100.0

### Measures

#### Socio-demographic questionnaire

There were six questions regarding participants’ gender, age, socioeconomic status, educational level, academic degree, and professional experience in the Socio-Demographic Questionnaire. We used this measure to collect the demographic characteristics of the participants.

#### Beck hopelessness scale

The BHS was developed by Beck et al. (1974) to determine the negative expectation level of an individual about the future. This self-assessment scale consists of 20 items with 11 true and nine false statements regarding an individual’s feelings and expectations about the future. The questions on the scale are answered in a right-wrong manner, and the scale reflects negative expectations. The obtained total score constitutes the “hopelessness” score ranging from 0 to 20. A higher score demonstrates a higher level of experienced hopelessness. Hopelessness scores obtained from the BHS are interpreted with the following cutoff points: 0–3 means a lower level of hopelessness, 4–8 is mild hopelessness, 9–14 is moderate hopelessness, and 15–20 is severe hopelessness (Beck and Steer, 1988). The scale consists of three dimensions: affective (feelings about the future), motivational (loss of motivation), and cognitive (expectations concerning the future). The scale has been shown to be internally consistent (*α* = 0.81; Kocalevent et al., 2017).

As the factor structure of the BHS may differ in clinical and nonclinical subjects as a function of nationality (Pompili et al., 2007), we performed confirmatory factor analysis (CFA) in the study sample, to investigate three competing models: (1) three-factor model, (2) an alternative two-factor model, and (3) another alternative one-factor model. We used weighted least squares to estimate the BHS factor models due to the multivariate non-normality of data and the dichotomous nature of variables. The adequacy of the model was assessed by a range of fit indices, namely the Root Mean Square Error of Approximation (RMSEA), Comparative Fit Index (CFI), and Tucker–Lewis Index (TLI) with 95% confidence intervals. CFI and TLI ratios ≥0.90, SRMR ≤0.08, and RMSEA ≤0.08 show acceptable fit, whereas good fit obtains CFI and TLI ≥0.95, SRMR ≤0.06, and RMSEA ≤0.06 (Hu and Bentler, 1999).

To test the three-factor model, items were set to load on the Affective, Cognitive, and Motivational factors to understand if items would converge in a three-factor structure as originally specified in the BHS (Beck et al., 1974). The results indicated a good model fit (RMSEA = 0.06, SRMR = 0.06, GFI = 0.94, CFI = 0.95, NFI = 0.93, TLI = 0.96). All items loaded significantly on their allocated factors. Examination of levels of factor loading indicated satisfactory factor loading of all items on the related factors (0.356–0.869) except for item 4 (0.196). Factor correlations between the Cognitive and Affective factors (0.89), the Cognitive and Motivational factors (0.78), and the Motivational and Affective factors (0.81) indicated poor differentiation.

To test the alternative two-factor model with two distinct content-related factors (i.e., hopelessness vs. hopefulness), as proposed by Szabó et al. (2016), we set all positively worded items to load on the Hopefulness factor and all negatively worded items on the Hopelessness factor. The results of the analysis exhibited acceptable model fit (RMSEA = 0.07, SRMR = 0.08, GFI = 0.91, CFI = 0.93, NFI = 0.91, TLI = 0.92). All items significantly loaded on their allocated factors (0.298–0.804) except for item 3 (0.187). There was also a high correlation between the factors (−0.87), indicating poor differentiation.

Finally, we assessed the one-factor model by allowing all items to load on one overall hopelessness factor. CFA of the one-dimensional model demonstrated a good model fit (RMSEA = 0.05, SRMR = 0.06, GFI = 0.97, CFI = 0.95, NFI = 0.93, TLI = 0.95). Compared to other models, this one-factor model exhibited better fit indices. As the correlations only marginally differ per factor in two-and three-factor models and due to a poor differentiation between factors, we used the one-dimensional approach to assess participants’ experienced hopelessness. The Kuder–Richardson reliability coefficient for the one-dimensional model was 0.71.

#### Multi-dimensional scale of perceived social support

This measure (Zimet et al., 1988) is a self-report questionnaire with 12 items that measure individuals’ perceptions of social support with three dimensions (i.e., family, friends, and significant others). Participants rate the sentences (e.g., “I can count on my friends when things go wrong”) on a 7-point Likert scale ranging from 1 (*very strongly disagree*) to 7 (*very strongly agree*). Total scores were calculated across items with higher scores denoting higher levels of perceived social support. The scale has demonstrated excellent internal consistency (*α* > 0.91) and good convergent validity (*r* = 0.48; Nearchou et al., 2019). Factor structure, reliability, and validity of the scale have also been examined in Iran, demonstrating acceptable values. The results of CFA in the study sample revealed adequate model fit (RMSEA = 0.06, SRMR = 0.05, GFI = 0.95, CFI = 0.93, NFI = 0.96, TLI = 0.94). The Cronbach’s alpha reliability value of the scale in the current study was 0.70.

### Procedures

Primarily, the researcher met with the department of education and public school administrators in several districts to find interested teachers and to ask them to complete and share the questionnaires with other teachers. The surveys had all the required instructions and informed consent to communicate voluntary participation. As the use of social media increased during the pandemic for teachers to communicate with each other and their students, we used a WhatsApp channel (a common social platform in Iran) to collect data. The administrators introduced the channel with members of teachers working at public schools in Tehran to the researcher, who were invited to participate in the study. This channel had 203 members of teachers, who were briefed about the purpose, procedure, and timeline of the study, as well as their rights and privacy. After responding to teachers’ inquiries, we shared the surveys in the channel to be completed. Of the 203 members of the group channel, only 168 (82%) teachers completed and returned the surveys through WhatsApp to the researcher.

The participating teachers were also paid for their time and collaboration in completing the surveys of the study. The results of the study were also shared with them at the end of data collection. The data collection procedure lasted almost a month and was completed in May 2021. Surveys with missing data or incomplete measures (*n* = 8) were returned to the participants to be completed again. The study was initiated in March 2021 and completed in June 2021.

### Data analysis

We used SPSS version 26 to analyze the data. In the analysis of the data, the arithmetic means and standard deviations were calculated, and the prevalence of hopelessness in the sample was reported in percentages. The Chi-square ( $\chi^{2}$ ) test was used to analyze differences between independent variables and hopelessness prevalence by forming double cross tables. Therefore, the sample was divided into “hopeless” and “not hopeless” teachers based on the cutoff values. A Kendall’s tau-b was conducted to investigate the relationship between perceived social support and hopelessness. We hypothesized that perceived social support should be negatively associated with hopelessness. Then, we conducted a binary logistic regression to test the potential effects of the demographic and occupational attributes on teacher hopelessness and to report odds ratios (OR) with the corresponding *p*-values and confidence limits.

## Results

### Prevalence of teacher hopelessness

The mean score on the hopelessness scale was 9.01 (SD = 5.92, *N* = 168), demonstrating a moderate hopelessness level in the population. The results indicated that 28 teachers (29%, *M* = 17.78, SD = 1.17) experienced severe hopelessness, 50 teachers (53%, *M* = 13.36, SD = 0.963) experienced moderate hopelessness, and 17 teachers (18%, *M* = 7.52, SD = 0.717) reported mild levels of hopelessness. Table 2 presents the results of Chi-square tests indicating the prevalence of experienced hopelessness in teachers. The prevalence of hopelessness was 56.5%, indicating a high occurrence of this mental health issue among teachers. The risk of being diagnosed with hopelessness as a function of gender was higher for males (79%, Relative risk = 2.07, 95% CI [1.56, 2.76]). There was also a significant difference between novice and experienced teachers in feelings of hopelessness. The prevalence and relative risk of hopelessness feelings were greater for the experienced teachers (82%, Relative risk = 2.06, 95% CI [1.59, 2.70]).

**Table 2:** Prevalence of hopelessness based on demographic and occupational attributes.

Attributes	Hopelessness				$\chi^{2}$	*p*	OR (95% CI)	φ	Total
	Yes		No						
	*n*	%	*n*	%					*n*, %
**Age groups**									
25–30	20	28.6	50	71.4					74, 44.0
30–35	34	77.2	10	22.7					42, 25.0
35–40	24	75.0	8	25.0	38.26	0.000	8.50 (2.76, 26.15)^1^	0.47	32, 19.0
>40	17	77.3	5	22.7	28.34	0.000	6.11 (3.05, 12.22)	0.41	20, 11.9
**Gender**									
Male	60	78.9	16	21.1					76, 45.2
Female	35	38.0	57	62.0					92, 54.8
**Education**									
B.A.	58	56.3	45	43.7	0.267	0.923	0.969 (0.37, 2.53)^3^	0.04	104, 61.9
M.A.	26	59.1	18	40.9					44, 26.2
Ph.D.	11	52.4	10	47.6					20, 11.9
**Education Level**									
Middle school	39	40.2	58	59.8	24.94	0.000	5.55 (2.75, 11.17)	0.38	97, 57.7
High school	56	78.9	15	21.1					71, 42.3
**Socioeconomic status**									
Low	62	69.7	27	30.3					91, 54.2
Middle	28	48.3	30	51.7	17.01	0.000	7.34 (3.82, 52.44)^2^	0.32	57, 33.9
High	5	23.8	16	76.2					20, 11.9
**Professional experience**									
Novice	40	39.6	61	60.4	29.59	0.000	6.99 (3.33, 14.66)	0.42	101, 60.1
Experienced	55	82.1	12	17.9					67, 39.9
**Perceived social support**									
Low	72	79.1	19	20.9	41.17	0.000	8.89 (4.40, 17.96)	0.49	91, 54.2
High	23	29.9	54	70.1					77, 45.8
**Total**	95	56.5	73	43.5					168, 100

1 Assessed based on 25–30 and > 40 age ranges.

2 Assessed based on low and high socioeconomic status.

3 Assessed based on B.A. and Ph.D. academic degrees.

Age was another significant factor associated with hopelessness feelings. The results indicated higher levels of hopelessness in older ages (>30), and the risk of hopelessness heightens with an increase in age. The odds ratio for age (25–30 and >40) and hopelessness feelings were quite large (OR = 8.50, 95% CI [2.76, 26.15]), representing a strong association between these variables. More precisely, the relative risk of hopelessness feelings for teachers over 40 years of age was 2.71 (95% CI [1.75, 4.17]) higher than for teachers within the 25–30 age range. Additionally, the prevalence of hopelessness was high for teachers with >40 (77%) and 30–35 (77%) age ranges. Regarding participating teachers’ education, we found no significant difference between groups (*p* > 0.05), indicating that the academic degree of the teachers was not associated with their hopelessness. In other words, the prevalence of hopelessness feelings among teachers with different educational degrees was approximately consistent (52%–59%). However, hopelessness feelings were different in teachers working in middle schools and teachers working in high schools. The prevalence and relative risk of hopelessness feelings were higher for teachers working in high schools (79%, Relative risk = 1.96, 95% CI [1.49, 2.57]) than for teachers working in middle schools (40%).

Another important factor associated with hopelessness was socioeconomic status, which revealed a strong relationship (OR = 7.34, 95% CI [3.82, 52.44]). Compared to teachers with high (24%) and middle (48%) socioeconomic status, experienced hopelessness was higher for teachers with low socioeconomic status (70%). In other words, the relative risk of experiencing hopelessness for teachers with poor socioeconomic status was 2.93 (95% CI [1.34, 6.36]) higher than for teachers with high socioeconomic status. Therefore, poor socioeconomic status could be a significant risk factor associated with hopelessness feelings in teachers. Similarly, there was a significant difference between groups of teachers in terms of perceived social support and the associated hopelessness feelings. As expected, the prevalence of hopelessness was greater for teachers with low perceived social support (79%) than teachers with high perceived social support (30%). The results indicated that the relative risk of being diagnosed with hopelessness as a function of perceived social support was 2.64 (95% CI [1.85, 3.79]) times greater for teachers with low perceived social support. The effect sizes for all significant associations in findings were moderate (Phi = 0.32–0.49).

### Effects of demographic and occupational attributes

Binomial logistic regression was performed to understand the effects of teachers’ demographic and professional attributes (independent variables) on the probability that participants may experience hopelessness feelings (outcome variable). The logistic regression model was statistically significant, $\chi^{2}\left(11\right)=125.46,p<0.001\text{.}$ Additionally, the model explained 70.6% (Nagelkerke *R*^2^) of the variance in hopelessness feelings and correctly classified 83.3% of cases.

The results indicated that gender significantly influences hopelessness feelings $\left(\beta =1.25,SE=0.53,p=0.02\right)$ by increasing the relative risk of hopelessness feelings for males, $\exp\left(B\right)=3.50,95\%CI\;\left[1.22,10.03\right]\text{.}$ In other words, the probability of experiencing hopelessness for males is 3.50 times higher than for females. The findings also indicated that teachers’ professional experience significantly predicts hopelessness feelings $\left(\beta =1.97,SE=0.91,p=0.03\right)\text{.}$ The odds of experiencing hopelessness were $\exp\;\left(B\right)=7.23\;\left(95\%CI\;\left[1.21,23.10\right]\right)$ times greater for experienced teachers as opposed to novice teachers.

Age was also a significant predictor of the prevalence of hopelessness in teachers within the 30–35 age range $\left(\beta =2.31,SE=0.85,p<0.001\right)$ and teachers over 40 years $\left(\beta =2.08,SE=1.34,p=0.04\right)$ . Compared to teachers with other age range (i.e., 25–30 and 35–40), the relative risk of experiencing hopelessness in these teachers were significantly high (exp(B) = 11.63, 95% CI [5.19, 37.07]), suggesting older age as a risk factor affecting teacher hopelessness. Regarding teachers’ education, there was a significant reduction in hopelessness levels of teachers with M.A. (*β* = –3.61, *SE* = 1.03, *p* < .001, exp(B) = .027, 95% CI [.004, .205]) and Ph.D. (*β* = –3.67, *SE* = 1.12, *p* = .001, exp(B) = .026, 95% CI [.003, .233]) academic degrees. The findings also indicated that high school teachers (*β* = 1.59, *SE* = .676, *p* = .018) were more susceptible to experience hopelessness than middle school teachers. The relative risk of experiencing hopelessness in high school teachers was exp(B) = 4.93 (95% CI [1.31, 18.54]) times greater compared to middle school teachers, suggesting a higher risk of hopelessness associated with being a high school teacher.

We also found significant effects of socioeconomic status on the likelihood of experiencing higher levels of hopelessness for teachers with low socioeconomic status (*β* = 2.15, *SE* = 0.882, *p* = 0.014). More precisely, the odds of experiencing hopelessness among teachers with low socioeconomic status were quite greater (exp(B) = 8.65, 95% CI [1.53, 38.76]) than teachers with middle (*β* = 1.34, *SE* = 0.985, *p* = 0.17) and high socioeconomic status. Similarly, the level of teachers’ perceived social support significantly accounted for the variation in hopelessness. Low perceived social support was associated with greater feelings of hopelessness (*β* = 2.05, *SE* = 0.547, *p* < 0.001) by increasing the odds of experiencing hopelessness up to 7.78 (95% CI [2.66, 22.72]) times greater than teachers with high perceived social support. Therefore, we may argue that low socioeconomic status and low perceived social support are two important risk factors influencing and increasing the relative risk of experiencing hopelessness feelings.

Furthermore, we performed Kendall’s tau-b to understand the significance of the correlation between perceived social support and hopelessness. As expected, the results indicated that perceived social support is negatively associated with hopelessness (*r_τ_* = −0.495, *p* < 0.001), suggesting that teachers with higher perceived social support are less likely to experience hopelessness feelings. Therefore, it can be argued that hopelessness is associated with contextual support; however, it may require much research and intensive interviews with the participants to determine its effect on teachers’ emotions and hopelessness.

## Discussion and implications

The results revealed that the teachers in this study experienced a moderate level of hopelessness. Additionally, the study demonstrated seven notable factors significantly associated with hopelessness. In particular, we found that the probability of moderate-to-severe hopelessness was about eight-and-a-half-fold higher when the financial situation was poor, nearly eight-fold higher when perceived social support was low, and about 11-fold higher with the increase in teachers’ age. Additionally, we found a negative association between teachers’ perceived social support and hopelessness, which may signify the importance of teachers’ emotions and emotional management in dealing with feelings of hopelessness.

Our results regarding a moderate level of experienced hopelessness are in line with previous studies with a general population (e.g., Haatainen et al., 2004; Hamzaoglu et al., 2010) and a university population (Poch et al., 2004). However, the results of the current study could have been affected by the COVID-19 pandemic circumstances, which have multiplied the challenges and stressors that teachers face by giving rise to unprecedented pandemic-specific problems (e.g., isolation and lockdown, social and physical distancing, health concerns, insomnia, loss of employment, income reduction, and emotional upset; see Holman et al., 2020; Baker et al., 2021; Ghasemi et al., 2022). As a result, the prevalence of hopelessness in teachers could have increased, similar to other mental illnesses (e.g., depression), due to the pandemic-related changes and modifications in teachers’ lives, teaching, and education.

Furthermore, the results indicated that male teachers were more prone to experience hopelessness feelings than female teachers, which was in line with previous studies (e.g., Haatainen et al., 2004; Lester, 2013). However, there are also some studies reporting no significant differences across gender (e.g., Hamzaoglu et al., 2010; Kocalevent et al., 2017), suggesting inconsistent results for the effects of gender on hopelessness feelings. This inconsistency may be the result of contextual, cultural, and ethnic differences between the study populations. Whether teachers work in middle or high schools was also a significant predictor of hopelessness in teachers. The prevalence of hopelessness was greater for high school teachers, which could be attributed to students’ misbehavior and learned helplessness in high schools in Iran (Ghasemi, 2022a). In other words, students’ disruptive and aggressive behaviors and lack of learning and achievement may culminate in loss of motivation, helplessness, and negative experiences for teachers, which may, in turn, lead to hopelessness feelings (Au et al., 2009; Chang et al., 2010). We also observed a trend of the increasing prevalence of hopelessness with age, which was consistent with previous studies (e.g., Hamzaoglu et al., 2010; Serin and Doğan, 2021). As age is positively associated with depression symptoms (Beekman et al., 1999; Snowdon, 2001) and psychological distress (Qiu et al., 2020), the probability of experiencing hopelessness may also increase with age due to the high correlation of hopelessness with these mental disorders (Au et al., 2009).

Another significant predictor of hopelessness was teachers’ socioeconomic status. The results indicated a greater odds of experiencing hopelessness for teachers with poor socioeconomic status. Our results are consistent with past findings indicating that a higher probability of experiencing hopelessness feelings is associated with people with low socioeconomic status and social class (e.g., Haatainen et al., 2004; Hamzaoglu et al., 2010). Evidence suggests that poor socioeconomic status during the pandemic could result in poor self-reported mental health (e.g., depression, anxiety, and psychological distress) in adults. As teachers in Iran live on low salaries and struggle to earn their living, their socioeconomic status may have been more complicated due to the lockdown and self-isolation during the pandemic, which may restrict their professional income and increase the odds of experiencing mental health issues.

Similar results were also found for perceived social support, which significantly accounted for variation in hopelessness. It has already been established that perceived social support may filter many psychological risk factors and promote mental health (Lai and Ma, 2016). According to the literature, high perceived social support has the potential to act as a protective factor against hopelessness during the pandemic (Zuo et al., 2021; Ghasemi, 2022b). Therefore, it is reasonable to assume that peer coaching and promoting social support for teachers diagnosed with hopelessness could improve their emotional and behavioral functioning (DeFronzo et al., 2001).

Furthermore, hopelessness could be caused by various sources (e.g., self, family, community, workplace climate, and colleagues), which may prevent or promote hopelessness and pessimism in teachers. If ignored and not properly treated, hopelessness can give rise to anxiety, burnout, depression, loneliness, and even suicidal ideation (Chang et al., 2010; Serin and Doğan, 2021). Therefore, administrators are encouraged to promote academic buoyancy (Huang, 2022), perceived social support (Cakar and Karatas, 2012; Zuo et al., 2021), and functional coping strategies (Ghasemi et al., 2022; Ghasemi, 2022b) to help at-risk teachers cope with hopelessness.

The results of the current study may help counselors, school psychologists, and administrators recognize risk factors related to hopelessness more effectively than earlier. For instance, they may consider those attributes significantly associated with hopelessness in their initial screening of hopelessness in public school teachers to prioritize therapeutic interventions for them. Additionally, the study provides evidence regarding the critical role of social support (e.g., collegial/superior support) and positive school climate as protective factors in alleviating hopelessness feelings. Findings support the expanded hopelessness theory of depression in the pandemic era and highlight the importance of social support by keeping personal relationships with family, friends, and colleagues alive when facing a mass trauma. In light of the presented results of the study, it is recommended that teachers are provided with knowledge of hopelessness, its consequences, and functional coping strategies to either prevent or treat such mental health issues. It is also suggested that collegial and superior support, as well as a positive school climate, should be promoted with the at-risk teachers to maintain their mental health. More importantly, teachers in public schools should also be supported financially, as the poor socioeconomic status may lead to several mental illnesses and diminish their performance.

Due to the significance of the demographic and occupational attributes of teachers in predicting feelings of hopelessness during the pandemic, scale developers may integrate these factors into the scales associated with teacher well-being to facilitate hopelessness diagnosis and screening. Given the mental and social consequences of the pandemic for teachers (e.g., stress, anxiety, depression, loneliness, and distance working), policymakers should tailor professional development programs in order to equip teachers with the required coping strategies to effectively respond to new emotional and professional demands during the crisis situations.

## Limitations

This study also had some limitations. First, the study was limited by its cross-sectional design; thus, causal inferences are not appropriate. Second, the study was limited by utilizing self-report techniques, giving rise to possible concerns regarding shared biases or common method variance. Future research may control common method bias among the scales by implementing a longitudinal design and utilizing qualitative techniques (e.g., behavioral observation and in-depth interview), which may further triangulate and enrich the findings. More importantly, any generalization of the study findings to teachers of other disciplines and contexts should be made cautiously because of the sampling strategy and significant differences between teachers working in the education system in Iran and teachers of other countries. Therefore, further research with other teachers working in western countries, particularly during the pandemic, is required to elaborate on the findings of this study.

Due to the lack of sufficient sample size, we failed to examine the measurement invariance of the BHS scale, which may obscure or bias true associations or differences. Therefore, it is difficult to ascertain that any differences in scale means are due to true differences. Additionally, evidence suggests that the factor structure of the BHS may be distorted due to method effects (Szabó et al., 2016). Flores-Kanter et al. (2022) recommend using the correlated trait-correlated method minus one approach to model the method effect on scales, as it is a powerful approach giving the trait factor an unambiguous meaning and preventing the anomalous results associated with fully symmetrical bi-factor modeling. Future studies may also apply this model to analyze the internal structure of the BHS.

As this study aimed at determining the prevalence of experienced hopelessness in teachers and examining the effects of different demographic and professional attributes on their hopelessness, this study is not sufficient to reveal the causal factors and situations that give rise to experienced hopelessness. Thus, in-depth interviews with teachers could contribute to our understanding of their perspectives by allowing them to describe the current situation in a more comprehensive and detailed way. Future research could be conducted utilizing the mixed-method research design by considering the relationships between hopelessness and different organizational variables such as conflict, burnout, peer relationships, and the teacher–student relationships.

## Conclusion

This study explored the prevalence of hopelessness among public school teachers in Iran, resulting in a moderate level of hopelessness. Additionally, the current study investigated the risk factors associated with hopelessness feelings during the COVID-19 pandemic and the effects of various demographic and occupational attributes on the hopelessness situation. The results indicated that all investigated demographic and occupational factors significantly associated with and influenced hopelessness feelings. However, we failed to find any significant association between teachers’ education and hopelessness. The results provide information to help counselors, school psychologists, and administrators identify the risk factors associated with teacher hopelessness in public schools in order to develop training programs to prevent hopelessness feelings, diminished performance, and attrition in teachers. Promoting social support and a positive school climate as well as supporting teachers financially during the pandemic could be some potential protective factors to maintain and foster teachers’ mental health.

## Data availability statement

The raw data supporting the conclusions of this article will be made available by the author, without undue reservation.

## Ethics statement

The studies involving human participants were reviewed and approved by Urmia University. The patients/participants provided their written informed consent to participate in this study.

## Author contributions

The author confirms being the sole contributor of this work and has approved it for publication.

## Conflict of interest

The author declares that the research was conducted in the absence of any commercial or financial relationships that could be construed as a potential conflict of interest.

## Publisher’s note

All claims expressed in this article are solely those of the authors and do not necessarily represent those of their affiliated organizations, or those of the publisher, the editors and the reviewers. Any product that may be evaluated in this article, or claim that may be made by its manufacturer, is not guaranteed or endorsed by the publisher.
